# 2SigFinder: the combined use of small-scale and large-scale statistical testing for genomic island detection from a single genome

**DOI:** 10.1186/s12859-020-3501-2

**Published:** 2020-04-29

**Authors:** Rui Kong, Xinnan Xu, Xiaoqing Liu, Pingan He, Michael Q. Zhang, Qi Dai

**Affiliations:** 1 College of Life Sciences, Zhejiang Sci-Tech University, Hangzhou, 310018 China; 2 College of Science, Hangzhou Dianzi University, Hangzhou, China; 3 College of Science, Zhejiang Sci-Tech University, Hangzhou, 310018 China; 4 Department of Biological Sciences, Center for Systems Biology, University of Texas at Dallas, Richardson, TX 75080 USA; 5 Division of Bioinformatics, Center for Synthetic and Systems Biology, TNLIST, Tsinghua University, Beijing, 100084 China

**Keywords:** Genomic island detection, Genomic signature, Small scale test, Large scale test, Boundary detection

## Abstract

**Background:**

Genomic islands are associated with microbial adaptations, carrying genomic signatures different from the host. Some methods perform an overall test to identify genomic islands based on their local features. However, regions of different scales will display different genomic features.

**Results:**

We proposed here a novel method “2SigFinder “, the first combined use of small-scale and large-scale statistical testing for genomic island detection. The proposed method was tested by genomic island boundary detection and identification of genomic islands or functional features of real biological data. We also compared the proposed method with the comparative genomics and composition-based approaches. The results indicate that the proposed 2SigFinder is more efficient in identifying genomic islands.

**Conclusions:**

From real biological data, 2SigFinder identified genomic islands from a single genome and reported robust results across different experiments, without annotated information of genomes or prior knowledge from other datasets. 2SigHunter identified 25 Pathogenicity, 1 tRNA, 2 Virulence and 2 Repeats from 27 Pathogenicity, 1 tRNA, 2 Virulence and 2 Repeats, and detected 101 Phage and 28 HEG out of 130 Phage and 36 HEGs in *S. enterica Typhi* CT18, which shows that it is more efficient in detecting functional features associated with GIs.

## Background

The diversity of bacteria has increased, and can adapt to environmental changes. The adaptability of these microorganisms is partly due to horizontal gene transfer (HGT). In 1990, Hacker et al. discovered some viral gene clusters from some *Escherichia coli* genomes, but no other closely related species were found, these viral gene clusters were named Pathogenic Islands (PAIs) [1]. PAIs can be divided into many types, including symbiotic islands, metabolic islands, secretory islands, and resistant islands. Generally, genomic islands (GIs) are used as a standard term to refer to a group of genes that are 10–200 kb in length after horizontal transfer. The area of horizontal transfer was originally called the GIs until the gene function was fully determined. Based on their gene function, a more specific term was provided for their basic use [2].

In the genomic era, the importance of GIs should be taken seriously. With new genomic sequencing technology, we aim to identify genomic regions of other species that are different from other species or strains. Generally speaking, the more relevant taxonomy is a method to identify genomic islands associated with functions [3, 4]. Such as, the genomic islands are associated with the secretion system, iron absorption function, secretion of toxins and adhesions, all of which increase the survival rate of pathogens in the host [5, 6]. Pathogens can initially regulate the detectability of chromosomes and exhibit different pathogenic phenotypes [7, 8]. GIs in bacteria induce many adaptation processes, such as metal resistance, antibiotic resistance, and secondary metabolic characteristics, thereby providing environmental and industrial benefits [9, 10]. Therefore, the identification of GIs in different genomes has been a key factor in the study of microbial evolution and function.

In large-scale comparative genomics, GIs have characteristics such as different sequence composition, direct flanking, migration-related genes, and tRNA genes, which should be explored and used to identify GIs [4, 11–13]. Genomic islands are scattered using a system model different from the host. Therefore, their differences can be determined by comparison with the differences of 16srrna [14]. Some detection algorithms have been developed: local alignment methods [15], and whole alignment methods [16]. These methods are based on multiple genomic alignments are inconsistent or unique, aligned with genomes that may be considered GIs and conservative regions. At the same time, several methods for constructing and applying multi-layer large-scale genome comparisons have been reported for complex situations. For example, MobilomeFINDER revealed that tRNA genes are shared across several related genomes. Mauve searches for genomic islands around homologous tRNA [17]. GI identification using this method is related to interrupted tRNAs, and genomic islands that do not have tRNA may be lost. The above question can be solved by MOSAIC, which are used to determine whether a strain-specific region should be inserted into the tRNA region [18]. However, we often incorrectly identify inversions and translocations as a strain-specific region. Another widely used GI prediction method is IslandPick [19]. For a simple genome, IslandPick can first select the optimal comparative gene without any prejudice, and then call Mauve for genome-wide comparison construction. IslandPick avoids duplication with help of rechecking Mauve’s alignment regions [20, 21]. The above algorithms are based on genomic comparison methods and can therefore be limited to using annotations or closely related but unavailable genomes. Since there are many genomes, the genome of the target species should be carefully selected [22].

In addition, some algorithms are also used to detect genomic islands based on the component of genome sequence. These algorithms can yield high efficiency and must distinguish anomalous regions from the remaining genomic biases because GI has a different sequence composition from the host. They are useful to quickly identify GIs in a genome or sequence and do not require additional genomes. Two to nine long oligonucleotide sequences and GC content are often defined as the component of genome sequence [11, 23–26]. Such as, abnormal G-C content and codon frequency deviations are calculated using PAI-Finder to detect GIs, and candidate PAIs are further evaluated using PAI-Finder to determine whether PAI-like regions partially or completely span GIs [27]. The PAI database (PAIDB) and PAI Finder are combined on one platform, where you can download annotated data and prediction information [28, 29].

Hidden Markov Model (HMM) helps to remove or detect abnormal regions containing component deviations [23, 30–32]. For example, SIGI-HMM has constructed an HMM model to eliminate ribosome regions with codon usage preferences [30, 31]. In addition, HMMer can identify the PFAM37 migrating gene map by searching each predicted gene [12], so IslandPath DIMOB [32] uses HMM to identify migrating gene map [33]. In contrast, Alien_Hunter improved the prediction of the boundaries of GIs by introducing a special scoring system based on k-mers variable length and using HMM models [23]. Although these methods based on Hidden Markov Models are more efficient than other methods in predicting GIs, they require a relatively large amount of parameter training and a large number of calculations. Therefore, prolonged operations are necessary to predict one GI.

A sequence is segmented into different regions, and the extraction of constituent characteristics of the sequence is performed instead of evaluating a set of genes in several predictions [34–37]. Measure significant differences between two windows to identify windows that are different in composition. The centroid method is used to determine some windows as GIs based on the comparison of windows’ scores [34]. But, it is limited by host signature estimates based on all windows. As a result, some noise was observed in the host’s local information. INDeGenIUS finds a cluster of the sequences to obtain a “major cluster” and estimates the host’s native signature. In this way, the previous problems can be solved [35, 36]. However, the measurement of each oligonucleotide is unnecessary, and some oligonucleotides are considered to be important indicators of horizontal transfer. Therefore, SigHunt detects the core tetranucleotides based on the related genomes using the tetranucleotide mass fraction instead of selecting all possible tetranucleotides [37].

Although the above algorithms achieve better performances, there are still some problems: 1) some methods mainly detect GIs through global testing, and pay attention to whether the local signature of a region is obviously not the same with the host. But, these characteristics are directly related to the scale of genomic signatures, for example, poor local genomic signatures may miss some small details at large scale; in contrast, small-scale features retain local features, whereas the GI detection is largely affected by large-scale differences. Therefore, the future developments of GI prediction should use multi-scale methods to explore the multi-scale genomic signatures; 2) the above algorithms detect some typical regions as possible genomic islands and do not refine the boundaries. If the predicted boundary of GIs can be further optimized, the effectiveness and efficiency of the prediction will be improved.

To address these problems, we proposed here a novel method “2SigFinder”, the first combined use of small-scale and large-scale statistical testing for genomic island detection. We propose an iterative of a small-scale t-test with large-scale feature selection techniques for each region of the genome to facilitate quantification of its compositional differences with the host, instead of calculating the distance or discrete interval cumulative score for each region. We used the higher moments of each tetranucleotide and designed an iteration of large-scale statistical testing with dynamic signals from small-scale feature selection to identify some multi-window segments; in addition, we split them into optimal distinct segments according to the CG-content bias and detect the genomic islands. At last, the CG-based segmentation method and the Markovian Jensen–Shannon divergence are used to optimize the boundaries of genomic islands.

## Results

### Comparison to the algorithms based on the windows for detecting GIs

We evaluated the effectiveness of our algorithm by detecting GI/non-GIs. Langille et al. constructed GI analysis data from 675 complete bacterial genomes. All genomes have a sufficient number of related species or strains, using strict but possibly flexible standards [19]. They identified some regions stored in all genomes as negative datasets and built a standard dataset to evaluate the efficient of genomic island detection methods. The data contains 771 genomic islands, referred to as GI, as well as 3770 non-genomic island fragments (non-GI), ranging in length from 8 kb to 31 kb. Since these GIs and non-GIs come from 118 genomes, the genomes of representative species come from the field of bacteria and archaea.

2SigFinder was used to classify GIs / non-GIs, where the transformed window is 1, the eye window is 5, the neighborhood size is 10, the long window is 50, 256 core features and 4 dynamic features are used, with 10 iterations and 0.05 standard error. Finally, the 3 kb “raw” genomic islands were used to find the genomic island boundary. Three published algorithms were also evaluated on the same dataset with default values [34, 35, 37]. When we used the SigHunt and INDeGenIUS methods, the significance level 0.05 test was selected to identify genomic islands, where DIAS was calculated based on all of the tetranucleotides.

The overall accuracy of the 2SigFinder was 85.16%, which achieved the best results, while the overall accuracy of the other methods was similar, ranging from 80 to 82% (Table 1). As for accuracy and recall, it is easy to find that the recall rate of 2SigFinder exceeds 45%, and no other methods. INDeGenIUS got a better precision, but its accuracy was lower (19.99%) [35]. The SigHunt’s performance did not meet expectations, and we infer that it predicts more genomic islands (758), and the average length of the predicted fragments is smaller (4670 bp) compared with other methods (number: 277–346, and average length: 13146–22,423 bp). These results indicates that 2SigFinder outperforms other algorithms in genomic island detection.

**Table 1:** Comparison of the window-based methods Centroid, INDeGenIUS, SigHunt, and the proposed 2SigFinder on classification of GIs/non-GI datasets. The precision, recall and overall accuracy of each method are calculated based on the number of overlapping nucleotides in both published GIs and predicted GIs

Method	Predicted GI			At Nucleotide Level (%)		
	Total Length	Total Number	Average Length	Accuracy	Precision	Recall
Centroid	5,573,339	320	17,417	82.37	61.35	27.63
INDeGenIUS	3,641,371	277	13,146	82.43	67.94	19.99
SigHunt	5,813,441	758	4670	80.54	51.00	23.95
**2SigFinder**	**7,758,374**	**346**	**22,423**	**85.16**	**66.59**	**49.05**

### Identification of genomic islands in *Pseudomonas aeru-ginosa* LESB58

We next evaluated the proposed method 2SigFinder on *P. aeruginosa* LESB58 genome, whose genomic islands have been explored widely [38–40]. There are currently 6 prophage gene clusters and 5 annotated pathogenicity islands in *P. aeruginosa* LESB58 [38, 41, 42].

**Fig. 1:**
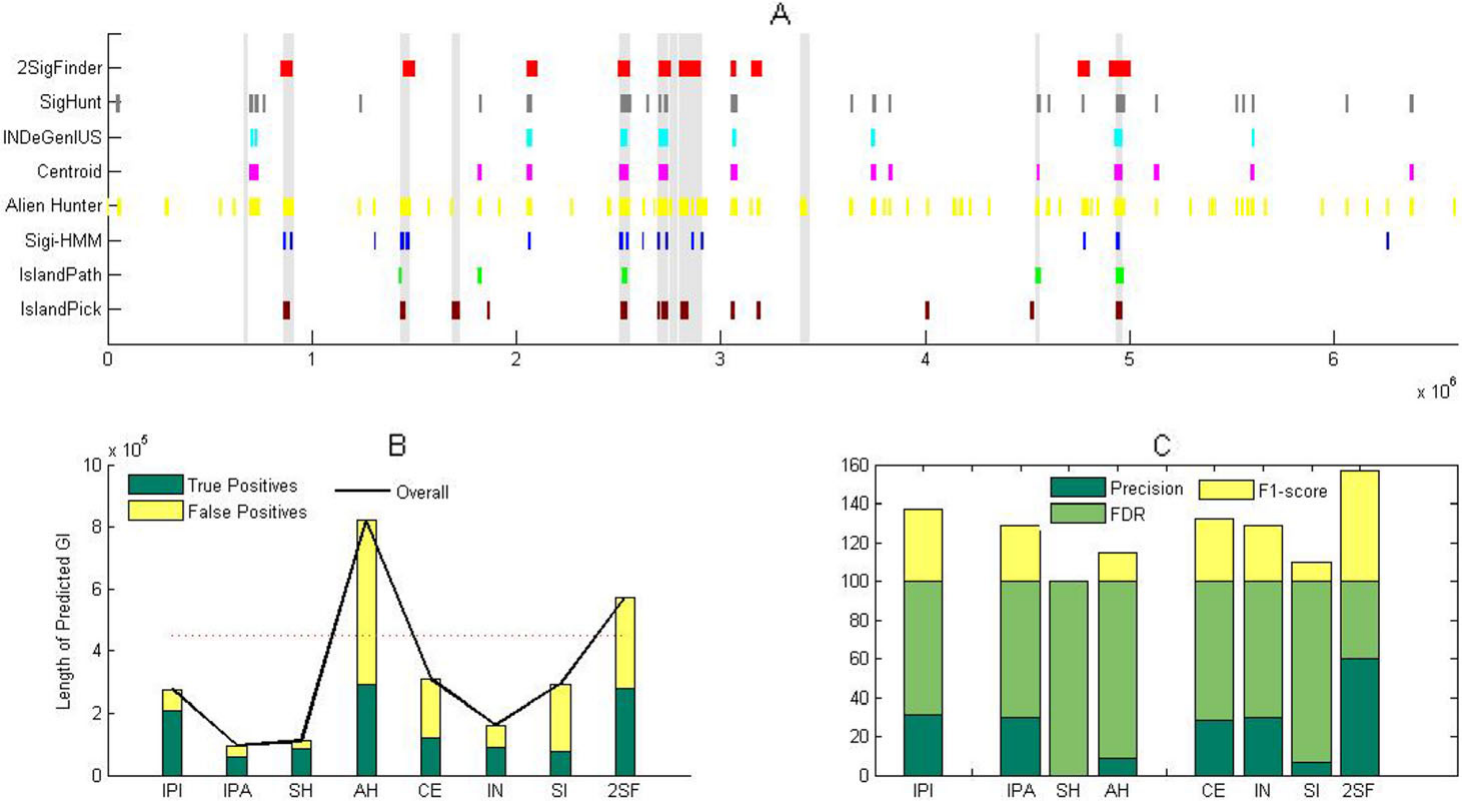
Performance of the proposed 2SigFinder (2SF), SIGI-HMM (SH), Al-ien_Hunter (AH), Centroid (CE), IslandPath-DIMOB (IPA), INDeGenIUS (IN), SigHunt (SI) and IslandPick (IPI) on the detection of genomic islands in P. aerugino-sa LESB58. **a** Predicted GIs found by all of the methods, and the known genomic islands are shown as vertical grey bars. **b** Overall length of the predicted genomic islands, true positives and false positives of all of the evaluated methods at the nucleo-tide level. **c** Precision, false positive rate (FPR) and F1-score of all of the evaluated methods at the island level, in which the precision, false positive rate and F1-score are calculated based on the number of known GIs that are more than 50% covered by the results of the prediction methods

We applied 2SigFinder to identify the genomic islands in the *P. aeruginosa* LESB58 genome, where transformed window is 4, eye window is 5, neighbourhood size is 4 and long window size is 100, using 256 core features and 4 dynamic features, with 4 iterations in IST-LFS and 4 iterations in ILST-DSFS, and 0.05 standard error. At last, 2 kb upstream/downstream of ‘raw’ genomic islands was used to refine the boundaries of predicted genomic islands. Six algorithms based on the windows and a comparative genomics were also used to predict the genomic islands with default values [19, 23, 31, 32, 34, 35, 37]. The level of the same significance test was set to 0.05, and the score results were used to identify the putative GIs. Figure 1a is the comparison of different detection algorithms on *P. aeruginosa*. LESB58 [37, 41, 42]. Since Alien_Hunter detected a large number of hypothetical regions, the predicted GI has the longest length (Fig. 1b). Note that although Alien_Hunter detected 293 kb in the established island-encoded 451 kb DNA, but its false positives was large (Fig. 1b). Thus, it gets the better recall at the expense of its accuracy (Fig. 1c and Tables 2 and 3).

**Table 2:** Total length, average length and number of genomic islands predicted by 2SigFinder, SIGI-HMM, Alien_Hunter, Centroid, IslandPath-DIMOB, INDeGenIUS, SigHunt and IslandPick on detection of genomic islands in *P. aeruginosa* LESB58, and total number of the overlapping nucleotides in both known GIs and predicted GIs Data as well as the number of the known GI with at least 50% covered by results of prediction methods

Method	Predicted GI			Nucleotides in both RGIs and PGIs^a^	RGIs/PGIs^b^ (> 50%)
	Length	Number	Average length		
IslandPick	275,178	16	17,199	209,001	5
IslandPath-DIMOB	95,919	10	9592	59,146	3
Sigi-HMM	110,465	21	5260	83,573	0
Alien Hunter	822,570	71	11,585	292,823	6
Centroid	308,000	14	22,000	121,503	4
INDeGenIUS	160,000	10	16,000	88,473	3
SigHunt	292,029	29	10,070	78,836	2
**2SigFinder**	**571,783**	**10**	**57,178**	**277,741**	**6**

^a^Total number of the overlapping nucleotides in both known GIs and predicted GIs Data

^b^Number of the known GI with greater than 50% covered by results of prediction methods

**Table 3:** Precision, false positive rate (FPR) and F1-score of the proposed method 2SigFinder, SIGI-HMM, Alien_Hunter, Centroid, IslandPath-DIMOB, INDeGenIUS, SigHunt and IslandPick on detection of genomic islands in *P. aeruginosa* LESB58, and the precision, false positive rate and F1-score are calculated based on the number of the known GIs with greater than 50% covered by results of prediction methods

Method		Method	Precision	FDR	F1-score
comparative genomics		IslandPick	31.25	68.75	37.04
Sequence composition	HMM-based methods	IslandPath-DIMOB	30	70	28.57
		Sigi-HMM	0	100	0
		Alien Hunter	8.45	91.55	14.63
	Window-based methods	Centroid	28.57	71.43	32
		INDeGenIUS	30	70	28.57
		SigHunt	6.90	93.10	10
		**2SigFinder**	**60**	**40**	**57.14**

In contrast, comparative genomics IslandPick got better prediction results by detecting 16 genomic islands. In order to further evaluate the predictive ability of GI level, we calculated the accuracy rate and F1 using the annotated genomic islands with more than 50% covered by the prediction results. Half of the 5 known genomic islands are predicted by IslandPick, which lead to high FDR and low F1 score (Fig. 1c and Tables 2 and 3).

2SigFinder predicted 10 genomic islands with large average length (Table 3). We observed that about 50% of the predicted 277,741 nucleotides were found in annotated genomic islands. It got a large true positive, and its false positive is also low (Fig. 1b). We then found that half of the 6 annotated genomic islands were predicted by 2SigFinder, resulting in the high accuracy and F1 (Fig. 1c and Table 3).

Through a comprehensive study, AlienHunter was found to be sensitive, but it has high false positive. Some algorithms based on the windows found some genomic islands, but their sizes are small. Thus, the results indicates that 2SigFinder is more efficient in identifying genomic islands.

### Identifying functional features in *S. enterica Typhi CT18*

Comparative genomics found that genomic island is often accompanied by different insertion sequences, repeat sequences and migratory tRNA genes. These features can better discover the function of genomic islands. Therefore, we further studied these functional features associated with the real genomic islands and predicted genomic islands from different prediction methods. We used the annotated genome to search for some characteristic genes in the genome islands. We looked for genes containing ribosomal proteins, genes with partner degradation functions, genes associated with energy metabolism, treated them as highly expressed genes, and counted their total number within genomic islands [39]. We used REPuter software to find repeated sequence fragments in genomic islands [40], and downloaded the annotation file from the US National Center for Biotechnology Information and looked for the insertion sequence within the genomic islands.

Here, we further analysed *S. enterica Typhi CT18* whose genomic islands was annotated [23, 43]. There are currently 17 pathogenicity islands in this sequence [23], and multiple phage has been found as well as the unidentified island [3, 44], resulted in 21 fragments reliably from foreign origin. All the functional features associated with genuine genomic islands have been summarized in Table 4.

**Table 4:** Summary of functional features predicted by 2SigFinder, SIGI-HMM, Alien_Hunter, Centroid, IslandPath-DIMOB, INDeGenIUS, SigHunt and IslandPick on detection of genomic islands in *S. enterica Typhi CT18*, and the functional features were based on the number of the related genes in the real genomic islands which are covered by more than 50% of the results of the prediction method

		Pathogenicity	Integrase	Phage	tRNA	HEG	Transposase	Virulence	Repeats	IS
Genuine GI		27	5	130	1	36	9	2	2	3
Predicted GIS	IslandPick	0	1	10	0	8	0	0	0	0
	IslandPath-DIMOB	0	2	58	0	10	1	0	0	0
	Sigi-HMM	16	0	5	0	6	3	0	0	0
	Alien_Hunter	**25**	**4**	65	1	23	6	2	2	3
	Centroid	4	0	3	1	3	0	2	1	0
	INDeGenIUS	5	2	1	1	7	0	2	0	0
	SigHunt	0	1	15	0	1	2	0	2	0
	**2SigFinder**	**25**	**3**	**101**	**1**	**28**	**2**	**2**	**2**	**2**

2SigFinder was used to detect genomic islands in this sequence, where transformed window is equal to 4, eye window size is 5, neighbourhood size is 4 and long window size is 100, using 256 core features and 4 dynamic features, with 8 iterations in IST-LFS and 10 iterations in ILST-DSFS, and 0.05 standard error. At last, it used 20 kb around genomic islands to search the GI’s boundary. Six algorithms based on the window and a comparative genomics were also used to predict the genomic islands with default values [19, 23, 31, 32, 34, 35, 37]. As before, we employed the same test with 0.05 level to detect the genome islands. All the functional features associated with the predicted genomic islands have been summarized in Table 4.

To evaluate the predicted GIs, we calculated their features within the real GIs, more than 50% of which was covered by the results of the prediction method. For Phage and HEG, 2SigFinder outperforms the other methods, and it detected 101 Phage and 28 HEG out of 130 Phage and 36 HEGs. As for features associated with GIs, including Pathogenicity, tRNA, Virulence and Repeats, 2SigHunter and Alien_Hunter achieve the best performance, where 25 Pathogenicity, 1 tRNA, 2 Virulence and 2 Repeats were identified from 27 Pathogenicity, 1 tRNA, 2 Virulence and 2 Repeats. For the Integrase, Transposase and IS features, Alien_Hunter outperforms the other methods. The next best method is 2SigFinder, whereas the other methods lag behind (Table 4).

**Fig. 2:**
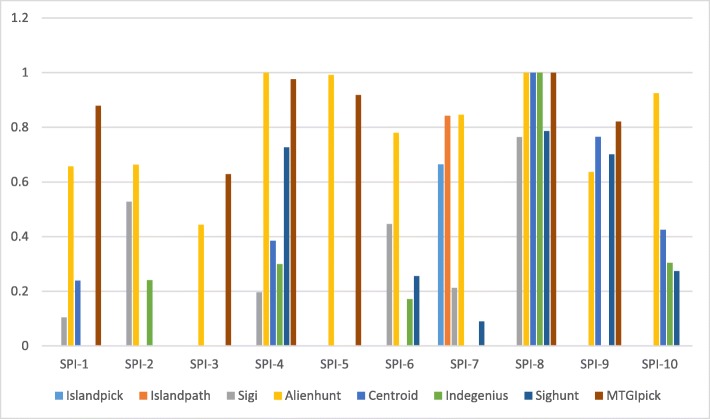
Overlap percentages between the reported PAI and the predicted genomic islands from Precision, recall and overall accuracy of SigHunt and INDeGenIUS, in which 0.05–0.2 significance levels are used as cut-off values to evaluate their performances. All evaluation indexes are calculated at the nucleotide level

PAI is a type of GIs that possesses the genetic elements of pathogens of virulence factors and affects the horizontal transfer of genes of multiple virulence factors. Ten PAIs are located in this genome as revealed by PAIDB [28, 29], and more information are summarised in Table 5. To further evaluate the predicted GIs, we counted the number of PAIs, more than 50% of which was covered by the results of the prediction method. Figure 2 indicates that Alien_Hunter achieves the best performance, with 9 out 10 PAIs were identified. The next best method is 2SigFinder, whereas the other methods lag behind. Moreover, Alien_Hunter performs better in detection of Integrase, Transposase, IS features and PAI because it predicted a lot of genomic islands, and its false positive is high (Table 6), indicating that it is of limited practical use. These results show that 2SigFinder is more efficient in detecting functional features associated with GIs.

**Table 5:** Ten pathogenicity islands reported to be located in *S. enterica Typhi CT18*, and name, star position, end position, size and function of these PAIs have been summarized from the pathogenicity island database (PAIDB)

Name	Pathogenicity islands			Function
	Star	End	Size(bp)	
SPI-1	2,858,736	2,900,586	41,851	Type III secretion system, invasion into epithelial cells, apoptosis (InvA, OrgA, SptP, SipA, SipB, SipC, SipD, SopE, prgH)
SPI-2	1,624,920	1,666,524	41,605	Type III secretion system, required for systemic infection and intracellular pathogenesis by facilitating replication of intracellular bacteria within membrane-bound Salmonella-containing vacuoles
SPI-3	3,883,613	3,900,553	16,941	Invasion, survival in monocytes, Mg2+ uptake (MgtC, B, MarT, MisL)
SPI-4	4,322,993	4,346,383	23,391	Type I secretion system, putative toxin secretion, apoptosis, required for intracellular survival in macrophages, genes weakly similar to RTX-like toxins
SPI-5	1,085,068	1,092,563	7496	Effector proteins for SPI-1 and SPI-2 (SopB, SigD, PipB)
SPI-6	302,092	360,757	58,666	safA-D and tcsA-R chaperone-usher fimbrialoperons6
SPI-7	4,409,511	4,543,148	133,638	Vi exopolysaccharide, SopE prophage and a type IVB pilus operon
SPI-8	3,132,530	3,139,414	6885	Two bacteriocin pseudogenes, genes conferring immunity to the bacteriocins
SPI-9	2,743,495	2,759,190	15,696	Type I secretory apparatus, large RTX-like protein
SPI-10	4,683,605	4,716,538	32,934	Phage 46 and the sefA-R chaperone-usher fimbrial operon

**Table 6:** Overall length of the predicted genomic islands, true positives and false positives of all of the evaluated methods at the nucleotide level in *S. enterica* Typhi CT18

Method	True positives	False positives	Overall length of PGIs
IslandPick	106,587	206	106,793
IslandPath	233,096	58,168	291,264
SIGI-HMM	137,308	103,846	241,154
Alien_Hunter	449,085	531,001	980,086
Centroid	68,483	105,517	174,000
INDeGenIUS	61,214	58,786	120,000
SigHunt	102,160	155,840	258,000
2SigFinder	357,218	97,551	454,769

PGI denotes predicted genomic islands

## Discussion

Genome islands refer to a type of gene clusters with horizontal origin in the genome, which is closely related to the rapid adaptation of the organism, making it have important values such as medical, economic or environmental. Comparative genomics analyses 16S rRNAs and other orthologs among different genomes to detect genomic islands. However, it relies largely on genomic comparison methods and thus can be limited to the use of annotations or closely related but unavailable genomes. Therefore, the emergence of research into comparison-free method is apparent and necessary to overcome critical limitations of comparative genomics.

Several algorithms have been proposed and achieve better performances, but there are still some problems in genomic island detection. 2SigFinder is a genomic island recognition method based on small-scale and large-scale statistical tests proposed by this paper. Through a comprehensive study, we found that AlienHunter was found to be sensitive, but it predicts more genomic islands, and the average length of the predicted fragments is smaller. Comparative genomics got better prediction results, but the number of genomic islands is predicted to be less. Some algorithms based on the windows found some genomic islands, but their sizes are small. 2SigFinder is more efficient in detecting genomic islands and their functional features. Although 2SigFinder achieved better performance, it is still not a generic solution to detect all GIs in different organisms. It relies on the observation of different tetranucleotides, thus only limited genomic signatures can be used. Sometimes, the detection of GI by tetranucleotide is not strong enough, which may lead to false negative prediction. For small genomic islands and not providing sufficient oligonucleotide patterns from their host genome, 2SigFinder may also be difficult to detect. Therefore, further research could also be conducted to determine genomic signatures that are more efficient for genomic island prediction.

## Conclusion

Several methods mainly detect GIs through global testing and pay attention to whether the local signature of a region is not the same with the host. In this paper, we proposed a genomic island recognition method based on small-scale and large-scale statistical tests. The existing methods generally have the predetermined thresholds, and the information of each window is limited. In the proposed method, we unique research the variability of higher moments of each tetranucleotide and designed an iteration of large-scale statistical testing with dynamic signals from small-scale feature selection to identify some multi-window segments; in addition, we split them into optimal distinct segments according to the CG-content bias. After depicting these compositionally different segments, the selection of genomic islands was performed by their IST-LFS scores. Finally, the CG-based divergence are used to optimize the boundaries of genomic islands. Systematic and quantitative assessment demonstrated that 2SigFinder is more robust than other existing methods in identifying genomic islands. As for the functional features associated with the real genomic islands, 2SigFinder is more efficient in inspection of the functions of genomic islands.

## Methods

**Fig. 3:**
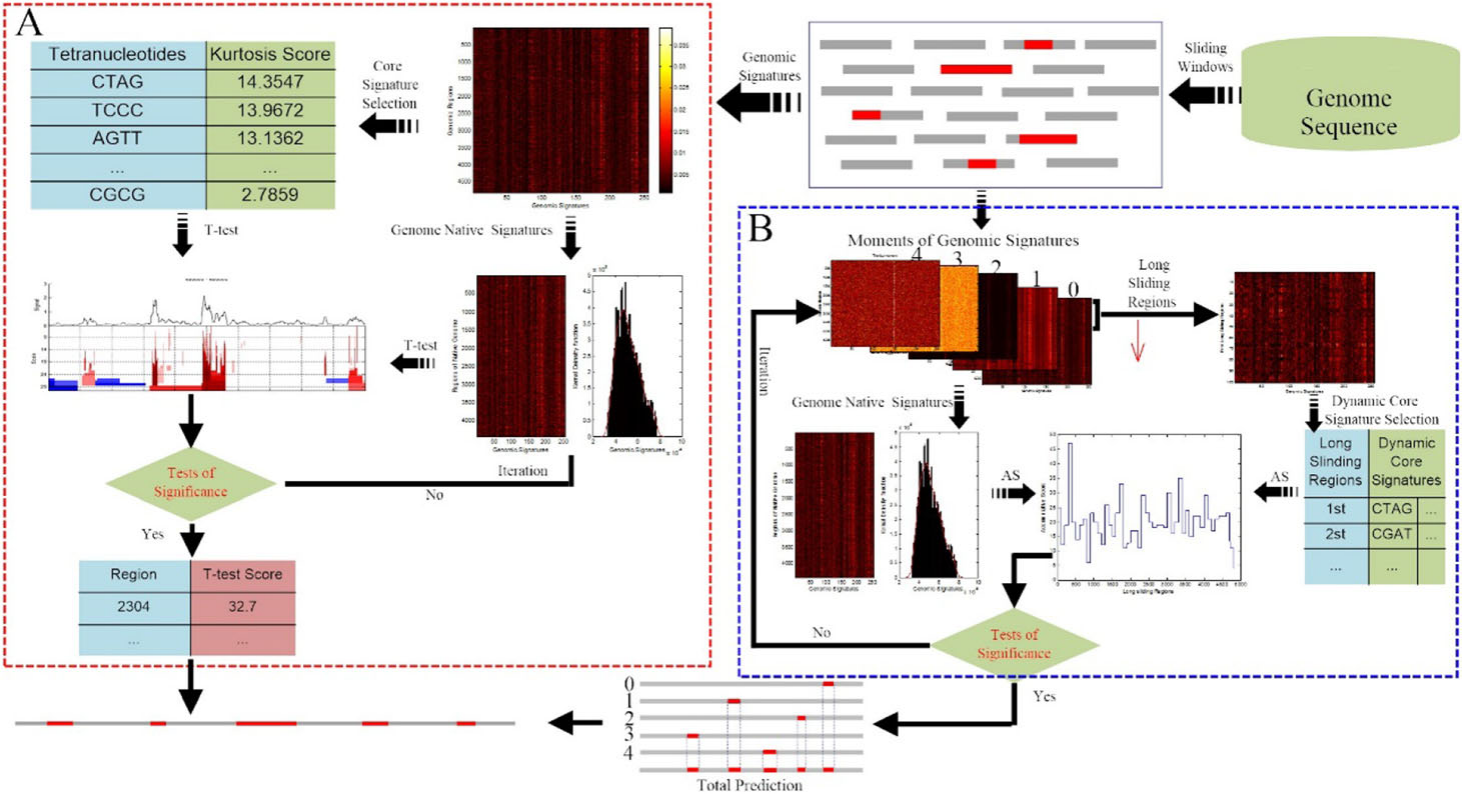
Overview of the 2SigFinder algorithm. **a** The work-flow of the small-scale t-test with large-scale feature selection, in which signatures of the host are extracted using the confidence interval of window variances, and core signatures are selected based on ordered kurtosis. During an iteration, we score each window using the two-sample t-test and selecte the windows whose scores are large enough to be considered to be statistically significant. **b** The workflow of the large-scale statistical test using dynamic signals from small-scale feature selection. Starting from the higher moments of each tetranucleotide, we select signatures of the host using the confidence interval of window variances and select dynamic core signatures using large sliding windows. During an iteration, we score each sliding long window with an accumulative score and select the windows whose scores are large enough to be consid-ered to be statistically significant

We designed a test-based algorithm to identify GI. The framework is shown in Fig. 3, and the steps are as follows:

At smaller scales, we used small-scale t-tests to score each window based on the large-scale selection to evaluate the component differences in each area (Fig. 3a). We first divided a genome into *n* windows with 1 kb long and calculated the frequencies *f* of the tetranucleotides. For each window, the confidence interval of the mean variance s2 was estimated as:

1 $$ \overline{s^2}-{z}_{\alpha /2}\frac{s_{s^2}}{N}\le {\mu}_{s^2}\le \overline{s^2}+{z}_{\alpha /2}\frac{s_{s^2}}{N} $$

where $\overline{s^2}$ is the mean value of all windows variances, ss2 is denoted as a variance, *α* is a confidence level, and *N* is the total number of the windows.

In *n* windows, the kurtosis of each tetranucleotide is defined as follows

2 $$ ku=\frac{\raisebox{1ex}{$\sum {\left({f}_i-\overline{f}\right)}^4$}\!\left/ \!\raisebox{-1ex}{$n$}\right.}{\raisebox{1ex}{${\left(\sum {\left({f}_i-\overline{f}\right)}^2\right)}^2$}\!\left/ \!\raisebox{-1ex}{$n$}\right.} $$

$\overline{f}$ is the average of a tetranucleotide. If a tetranucleotide has a larger kurtosis, it will be selected as the information signatures.

Given the *i*th window, we calculated the two-sample t-test between the host and the *i*th window. For each *f*_*j*_ of the *i*th window, we choose its left and right window regions as a sample $\left({f}_j^{i-\varepsilon +1},\cdots, {f}_j^i,\cdots, {f}_j^{i+\varepsilon}\right)$ of the signature *f*_*j*_ from the *i*th window. The signature *f*_*j*_ from the host was represented as $\left({f}_j^{t_1},{f}_j^{t_2}\cdots, {f}_j^{t_{\Gamma}}\right)$, and *t*_Τ_ tT is the window number from the host and Γ denotes the chose signatures. Then, we used the t-test to determine if the average values of the two samples $\left({f}_j^{i-\varepsilon +1},\cdots, {f}_j^i,\cdots, {f}_j^{i+\varepsilon}\right)$ fji-ε + 1,⋯,fji,⋯,fji + ε and $\left({f}_j^{t_1},{f}_j^{t_2}\cdots, {f}_j^{t_{\Gamma}}\right)$ fjt1,fjt2,⋯,fjtΓ are equal, and calculated the *P*-value of informative signature as follows:

3 $$ {P}_{f_j}=P\left(\left|t\right|>\frac{\overline{f_j^1}-\overline{f_j^2}}{\sqrt{s_p^2\left(\frac{1}{2\varepsilon +1}+\frac{1}{t_{\Gamma}}\right)}}\right) $$

where

$$ {s}_p^2=\frac{2\varepsilon {s}_{f_j^1}^2+\left({t}_{\Gamma}-1\right){s}_{f_j^2}^2}{2\varepsilon +{t}_{\Gamma}-1} $$

$\overline{f_j^1}$× 1 and $\overline{f_j^2}$ × 1 (${s}_{f_j^1}^2$ s12 and ${s}_{f_j^2}^2$) denote the average (variances) of the *i*th region fji-ε + 1,⋯,fji,⋯,fji + εand the host. Accumulating all the signature *p* values, the difference was as follows:

4 $$ D={\sum}_{j=1}^{t_{\Gamma}}{P}_{f_j} $$

Then we selected some windows with scores large enough to make the data statistically significant, and delete these selected windows. We updated all windows in the genome, and then repeated the above steps until no windows were found.

### A large-scale statistical test using dynamic signals from small-scale feature selection

On a large scale, we study the variability of the high-order moments of each tetranucleotide and use dynamic signals selected by small-scale features to design iterations of large-scale statistical tests to identify large, multi-window segments (Fig. 3b).

To assess changes of local signatures surrounding the *i*th window, we choose 2τ window surrounding the *i*th window as its neighbourhood and calculate the normalised first, second, third and fourth standardized moments of each signature as follows:

5 $$ {\mathrm{NM}}_{\mathrm{i}}^1\left({\mathrm{f}}_{\mathrm{t}}^{\mathrm{i}}\right)=\frac{1}{2\uptau +1}{\sum}_{\mathrm{x}=\mathrm{i}-\uptau}^{\mathrm{i}+\uptau}{\mathrm{f}}_{\mathrm{x}}^{\mathrm{i}} $$

6 $$ {\mathrm{NM}}_{\mathrm{i}}^2\left({\mathrm{f}}_{\mathrm{t}}^{\mathrm{i}}\right)=\sqrt{\frac{1}{2\uptau +1}{\sum}_{\mathrm{x}=\mathrm{i}-\uptau}^{\mathrm{i}+\uptau}{\left({\mathrm{f}}_{\mathrm{x}}^{\mathrm{i}}-{\mathrm{NM}}_{\mathrm{i}}^1\left({\mathrm{f}}_{\mathrm{t}}^{\mathrm{i}}\right)\right)}^2} $$

7 $$ {\mathrm{NM}}_{\mathrm{i}}^3\left({\mathrm{f}}_{\mathrm{t}}^{\mathrm{i}}\right)=\frac{2\uptau +1}{2\uptau \left(2\uptau -1\right)}{\sum}_{\mathrm{x}=\mathrm{i}-\uptau}^{\mathrm{i}+\uptau}{\left(\frac{{\mathrm{f}}_{\mathrm{x}}^{\mathrm{i}}-{\mathrm{NM}}_{\mathrm{i}}^1\left({\mathrm{f}}_{\mathrm{t}}^{\mathrm{i}}\right)}{{\mathrm{NM}}_{\mathrm{i}}^2\left({\mathrm{f}}_{\mathrm{t}}^{\mathrm{i}}\right)}\right)}^3 $$

8 $$ {\mathrm{NM}}_{\mathrm{i}}^4\left({\mathrm{f}}_{\mathrm{t}}^{\mathrm{i}}\right)=\frac{\left(2\uptau +1\right)\left(2\uptau +2\right)}{2\uptau \times \left(2\uptau -1\right)\left(2\uptau -2\right)}{\sum}_{\mathrm{x}=\mathrm{i}-\uptau}^{\mathrm{i}+\uptau}{\left(\frac{\mathrm{f}{}_{\mathrm{x}}{}^{\mathrm{i}}-{\mathrm{NM}}_{\mathrm{i}}^1\left({\mathrm{f}}_{\mathrm{t}}^{\mathrm{i}}\right)}{{\mathrm{NM}}_{\mathrm{i}}^2\left({\mathrm{f}}_{\mathrm{t}}^{\mathrm{i}}\right)}\right)}^4-\frac{24{\uptau}^3}{\left(2\uptau -1\right)\left(2\uptau -2\right)} $$

where ${\mathrm{NM}}_{\mathrm{i}}^1\left({\mathrm{f}}_{\mathrm{t}}^{\mathrm{i}}\right)$, ${\mathrm{NM}}_{\mathrm{i}}^2\left({\mathrm{f}}_{\mathrm{t}}^{\mathrm{i}}\right)$, ${\mathrm{NM}}_{\mathrm{i}}^3\left({\mathrm{f}}_{\mathrm{t}}^{\mathrm{i}}\right)$ and ${\mathrm{NM}}_{\mathrm{i}}^4\left({\mathrm{f}}_{\mathrm{t}}^{\mathrm{i}}\right)$ are the normalised first, second, third and fourth standardized moments of the signature ${\mathrm{f}}_{\mathrm{t}}^{\mathrm{i}}$ within the *i*th window.

We calculated the genomic signatures of the host and estimate the cumulative kernel distribution function φ for each signature. From the *i*th window, we use its following δ continued windows to create the *i*th large sliding window (LSW_i_ LSW_i_). We then select core signatures of these δ continued windows within the *i*th large windows using ordered kurtosis. It is important to highlight here that the core signatures of the large window will change as the *i*th window sliding along genome, and thus, we denote this set of core signatures as dynamic core signatures of this genome.

Count the top θ dynamic core signatures whose values are located outside of their credibility interval in non-overlapping windows, and sum all count numbers of the δ continued windows as accumulative score (AS) of the *i*th large sliding window

9 $$ \mathrm{AS}\left({\mathrm{LSW}}_{\mathrm{i}}\right)=\sum \limits_{\mathrm{i}=1}^{\updelta}\sum \limits_{\mathrm{t}=1}^{\uptheta}\mathtt{\varphi}\left({\mathrm{f}}_{\mathrm{t}}^{\mathrm{i}}\right) $$

Where $\mathtt{\varphi}\left({\mathrm{f}}_{\mathrm{t}}^{\mathrm{i}}\right)$ is a random indicator function defined as follows:

10 $$ \mathtt{\varphi}\left({\mathrm{f}}_{\mathrm{t}}^{\mathrm{i}}\right)=\left\{\begin{array}{cc}0& {\mathrm{f}}_{\mathrm{t}}^{\mathrm{i}}\in \left({\upvarphi}_{\mathrm{t}}^{-1}\left(\frac{\upalpha}{2}\right),{\upvarphi}_{\mathrm{t}}^{-1}\left(1-\frac{\upalpha}{2}\right)\right)\\ {}1& \mathrm{Otherwise}\end{array}\right. $$

φ_t_ φ_t_ is the cumulative kernel distribution function of the dynamic core signature f_t_, ${\mathrm{f}}_{\mathrm{t}}^{\mathrm{i}}$ is the value of the dynamic core signature in the *i*th non-overlapping window, and α is a confidence level.

Select large sliding windows whose scores are large enough to be considered statistically significant. Delete the selected large sliding window and update the entire window of the genome, repeating the steps above until the large sliding window cannot be found.

### Refine the boundaries of predicted GIs

For each multi-window region detected by the above method, we segment it into several different fragments based on the GC content deviation, and use the G-C deviation and Markovian Jensen-Shannon divergence (MJSD) to determine the boundaries of the predicted GIs. Assume *t*_1_ t1 and *t*_2_ are the start and end points of a given genomic island ${S}_{\left[{t}_1\to {t}_2\right]}$ St1 → t2. We search its boundaries from the expanded region ${S}_{\left[{t}_1-\gamma kb\to {t}_2+\gamma kb\right]}$ St1-γkb → t2 + γkb. G-C deviation is one of the important sequence features, describing the differences between DNA fragments [45, 46]. In order to find the starting position, the sequence St1-γkb → t2 is divided into different sub-sequences to get some points $\left\{{P}_{S_{\left[{t}_1-\gamma kb\to {t}_2\right]}}^{CG}\right\}$. For each point *t*_*τ*_, its MJSD was calculatedStτ→t2 as follows:

11 $$ {\displaystyle \begin{array}{l}{MJSD}^2\left({t}_{\tau}\right)={H}^2\left({S}_{\left[{t}_1-\gamma kb\to {t}_2\right]}\right)-\frac{t_{\tau }-{t}_1-\gamma kb+1}{t_2-{t}_1-\gamma kb+1}{H}^2\left({S}_{\left[{t}_1-\gamma kb\to {t}_{\tau}\right]}\right)\\ {}\kern10em -\frac{t_2-{t}_{\tau }+1}{t_2-{t}_1-\gamma kb+1}{H}^2\left({S}_{\left[{t}_{\tau}\to {t}_2\right]}\right)\end{array}} $$

where H2St1-γkb → tτ and H2Stτ → t2are the entropies of the ${S}_{\left[{t}_1-\gamma kb\to {t}_{\tau}\right]}$ and ${S}_{\left[{t}_{\tau}\to {t}_2\right]}$ respectively, H2St1-γkb → t2 is the entropy of St1-γkb → t2.

## Data Availability

Datasets and supplementary are freely available at https://github.com/bioinfo0706/2SigFinder or http://bioinfo.zstu.edu.cn/2SigFinder.

## Abbreviations

HGT: Horizontal Gene Transfer
PAI: Pathogenicity Island
GI: Genomic Island
PAIDB: PAI Database
HMM: Hidden Markov Model
CG-MJSD: GC content and Markovian Jensen-Shannon divergence
HEGs: Highly expressed genes
RP: Ribosomal protein
TF: Transcriptional processing factor
CH: Chaperone degradation
IS: Insertion sequence elements
NCBI: National Center for Biotechnology Information
